# Bypass Mechanisms of the Androgen Receptor Pathway in Therapy-Resistant Prostate Cancer Cell Models

**DOI:** 10.1371/journal.pone.0013500

**Published:** 2010-10-19

**Authors:** Rute B. Marques, Natasja F. Dits, Sigrun Erkens-Schulze, Wytske M. van Weerden, Guido Jenster

**Affiliations:** Department of Urology, Josephine Nefkens Institute, Erasmus Medical Center, Rotterdam, The Netherlands; Baylor College of Medicine, United States of America

## Abstract

**Background:**

Prostate cancer is initially dependent on androgens for survival and growth, making hormonal therapy the cornerstone treatment for late-stage tumors. However, despite initial remission, the cancer will inevitably recur. The present study was designed to investigate how androgen-dependent prostate cancer cells eventually survive and resume growth under androgen-deprived and antiandrogen supplemented conditions. As model system, we used the androgen-responsive PC346C cell line and its therapy-resistant sublines: PC346DCC, PC346Flu1 and PC346Flu2.

**Methodology/Principal Findings:**

Microarray technology was used to analyze differences in gene expression between the androgen-responsive and therapy-resistant PC346 cell lines. Microarray analysis revealed 487 transcripts differentially-expressed between the androgen-responsive and the therapy-resistant cell lines. Most of these genes were common to all three therapy-resistant sublines and only a minority (∼5%) was androgen-regulated. Pathway analysis revealed enrichment in functions involving cellular movement, cell growth and cell death, as well as association with cancer and reproductive system disease. PC346DCC expressed residual levels of androgen receptor (AR) and showed significant down-regulation of androgen-regulated genes (p-value = 10^−7^). Up-regulation of VAV3 and TWIST1 oncogenes and repression of the DKK3 tumor-suppressor was observed in PC346DCC, suggesting a potential AR bypass mechanism. Subsequent validation of these three genes in patient samples confirmed that expression was deregulated during prostate cancer progression.

**Conclusions/Significance:**

Therapy-resistant growth may result from adaptations in the AR pathway, but androgen-independence may also be achieved by alternative survival mechanisms. Here we identified TWIST1, VAV3 and DKK3 as potential players in the bypassing of the AR pathway, making them good candidates as biomarkers and novel therapeutical targets.

## Introduction

Prostate cancer (PCa) is the second leading cause of male cancer deaths in the Western countries and an increasing problem in those adopting Western lifestyle and diet. Advances in screening and diagnosis have allowed the detection of tumors at earlier stages, when curative therapy is still feasible. For late stage disseminated disease however, current therapies are merely palliative and no curative treatment exists. Since the growth of prostate tumors is originally androgen-dependent, metastatic cancers are generally treated with androgen ablation therapy, with or without antiandrogen supplementation [1], [2]. The vast majority of these patients show a significant clinical regression, but the cancer eventually recurs within 12–18 months. These recurrent tumors have escaped androgen suppression and became resistant to hormonal therapy, referred to as hormone-refractory or castration-resistant PCa. To survive and resume growth in an androgen-deprived environment PCa cells must either adapt the androgen receptor (AR) pathway to the androgen-depleted conditions or invoke alternative survival and growth pathways [3]. Much experimental evidence exists to support both mechanisms, which are not necessarily mutually exclusive. AR expression was shown to be maintained in the majority of patients that underwent hormonal therapy and showed recurrence of disease, suggesting a role of the AR also in late stage disease [4], [5]. Moreover, the AR gene is amplified and/or overexpressed in about 30% of the hormone-therapy refractory tumors, and it has been proposed this could sensitize the receptor for the residual androgen concentrations and antiandrogens present under hormonal therapies [6], [7], [8]. Furthermore, several AR mutations, resulting in increased activity or broadened ligand-specificity to alternative steroids and antiandrogens, have been associated with disease progression [9], [10]. Other modifications of the AR pathway that may induce hormone-refractory growth include intratumoral steroidogenesis, ligand-independent activation by cross-talk with other signaling pathways, alterations in the balance of AR co-regulators or expression of constitutively active truncated AR isoforms [3], [11], [12]. Interestingly, recent work from others and us has revealed that the AR pathway may be selectively attenuated in advanced/metastatic disease [13], [14], [15]. Since the AR pathway is also involved in processes of cellular differentiation and prostate maturation, it is tempting to suggest that PCa cells may eventually gain growth advantage by inhibiting the AR induced differentiation. Prompted by these results, we focused the present study on alternative survival and growth pathways, which are independent of AR activation. To effectively bypass the AR pathway, cancer epithelial cells must be able to survive the apoptotic signals triggered by hormonal therapies and invoke alternative growth pathways. Autocrine production of growth factors or its receptors, activation of oncogenes and inhibition of tumor-suppressor genes are all possible mechanisms for bypassing the AR pathway. Consistent with this hypothesis, paracrine growth factors that are normally secreted by prostate stroma cells, such as epidermal growth factor (EGF), insulin-like growth factor 1 (IGF1), hepatocyte growth factor (HGF), keratinocyte growth factor (KGF) or interleukin 6 (IL6), are found to be overexpression in hormone-refractory cancer in association with a switch to autocrine production by cancer epithelial cells [16]. In addition to being potential mitogens, mounting evidence indicates that these growth hormones are also able to cross-talk with the AR signaling pathway, leading to expression of AR target genes in the absence of androgens [17]. Therefore, it still has to be established whether the autocrine production of these growth factors represents an adaptation or a true bypass of the AR signaling pathway. Alterations in the anti-apoptotic *BCL2* oncogene and in the pro-apoptotic *P53* and *PTEN* tumor-suppressor genes have also been found in prostate cancer [18], [19]. However, these events mostly occur before late stage progression, making them less likely candidates for the switch to hormone-refractory growth in late stage disease. Nevertheless, by inhibiting PCa cell death and shifting the balance towards cellular proliferation, genes involved in the regulation of apoptosis may also play a role in hormone-refractory growth.

To explore the mechanism(s) by which androgen-dependent PCa cells become resistant to hormonal therapy, we used microarray technology to interrogate the differences in gene expression between androgen-responsive and therapy-resistant cell lines. As model system we used the androgen-responsive PC346C cell line and its therapy-resistant sublines PC346DCC, PC346Flu1 and PC346Flu2. These sublines were derived from the parental PC346C by long-term androgen ablation (PC346DCC), supplemented with the antiandrogen hydroxyflutamide (PC346Flu1 and PC346Flu2) [20], [21]. Previous studies revealed distinct AR modifications in all three therapy-resistant sublines, which corresponded to diverse mechanism of hormone-refractory growth. Whereas PC346DCC, expressing very low levels of AR and PSA, showed evidence of bypassing of the AR pathway, PC346Flu1 exhibited 4-fold AR up-regulation and PC346Flu2 was shown to carry the T877A AR mutation. Both PC346Flu1 and PC346Flu2 sublines replicate the progression to hormone-therapy refractory disease through adaptations of the AR pathway. Therefore, we focused on the PC346DCC subline to select for genes particularly involved in the bypass of the AR pathway. In addition to providing novel insights into the mechanisms of PCa progression, the genes identified here may prove useful as prognostic markers and potential targets for novel therapeutical approaches.

## Methods

### Reagents and cell lines

The PC346C cell line was derived from the prostate tumor of a patient with non-progressive prostate adenocarcinoma (T4N0M0) [20], [21]. The PC346DCC, PC346Flu1 and PC346Flu2 sublines were derived from PC346C upon long-term culture in charcoal-stripped medium, without or with antiandrogen hydroxyflutamide supplementation, respectively. The development and characterization of these cell lines has been published previously [20], [21]. The basic culture medium used in the maintenance of PC346 cell lines consisted of DMEM-F12 medium (Cambrex BioWhitaker, Belgium) supplemented with 2% fetal calf serum (FCS; PAN Biotech GmbH, Aidenbach, Germany), 1% insulin-transferrin-selenium (Gibco BRL), 0.01% bovine serum albumin (Boehringer Mannheim, Germany), 10 ng/ml epidermal growth factor (Sigma-Aldrich), penicillin/streptomycin antibiotics (100 U/ml penicillin, 100 mg/ml streptomycin; BioWhitaker, Belgium); plus the following additions: 100 ng/ml fibronectin (Harbor Bio-Products, Tebu-bio, The Netherlands), 20 mg/ml fetuine (ICN Biomedicals, The Netherlands), 50 ng/ml choleratoxin, 0.1 mM phosphoethanolamine, 0.6 ng/ml triiodothyronine and 500ng/ml dexametason (all from Sigma). PC346C cells were maintained in culture in the complete medium described above, supplemented with 0.1 nM 17-methyltrienolone (R1881; NEN, Boston MA, USA). PC346DCC selection medium was supplemented as described above, but depleted from androgens by using dextran-coated charcoal (DCC) treated FCS. PC346Flu1 and PC346Flu2 culture medium was also androgen depleted by using 2% DCC-FCS, and supplemented with 1 µM of hydroxyflutamide (OH-flutamide, Schering-Plough Research Institute, New Jersey, USA).

Cells were grown in T25 Primaria™ tissue culture flasks (BD Biosciences Benelux N.V, The Netherlands) at 37°C under 5% CO_2_ humidified atmosphere.

### Expression microarray analysis

Cells were seeded in their respective selection medium to reach ∼50% confluency and allowed to grow for 2 days. Then, cells were rinsed twice with PBS and stored at −20°C until RNA isolation. Total RNA was isolated with RNAzol B reagent (Campro Scientific, Veenendaal, The Netherlands) and further purified through RNeasy columns (Qiagen) with on-column DNA digestion, according to the manufacturer's protocol. RNA quality was checked on 1% agarose gel.

Cy3- or Cy5-labelled RNA probes were produced by incorporating amino-allyl UTP during RNA amplification, followed by coupling to N-hydroxysuccinimide modified dye. Briefly, 3 µg RNA was used for a T7-based linear mRNA amplification protocol, described previously [22]. Amino-allyl UTP, plus equal amount of unmodified rUTP, was incorporated into aRNA with T7 Megascript Kit (all from Ambion), according to manufacturer's protocol. Amplified RNA was purified and concentrated using Microcon-YM 30 columns (Amicon®) to rinse three times with 300 µl RNAse-free water. Finally, 2 µg aminoallyl-modified RNA, in a maximum of 3.33 µl of RNAse-free water, was incubated with 1.66 µl sodium bicarbonate buffer (0.3 M, pH 9) and 5 µl Cy3- or Cy5- dye (CyScribe Post-Labeling Kit, Amersham, NJ, USA), for 1 h in the dark at room temperature. Reaction was stopped with 5 µl 4 M hydroxylamine HCl (Sigma), contra-labelled probes were combined and purified/concentrated using Microcon-YM 30. Probe was collected in 5–15 µl final volume and resuspended in 80 µl Ambion hybridization buffer number 1.

For the microarray we used double-dye oligoarrays representing about 15,000 human genes, on which labelled RNA from the each therapy-resistant subline was cohybridized with contra-labelled PC346C. Four microarrays were performed per condition, using two distinct cell passages in dye-swap. This was done to account for the biological variability and to exclude dye-preferential binding to oligonucleotides on the microarray. The oligoarrays used in this study were produced at the Erasmus Center for Biomics. Briefly, a human 18,584 oligonucleotides library (Compugen, Sigma-Genosys) was spotted on aminosilane slides using a Virtek Chipwriter Professional arrayer (Virtek Vision International, Waterloo, Canada). Control spots included landmarks, spotting buffer, alien oligonucleotides (SpotReport Alien Oligo Array, La Jolla, Stratagene), poly d[A]40–60, salmon sperm DNA, and human COT-1 DNA. Before the hybridization, microarray slides were prehybridized in 5x SSC, 0.05% SDS, 4% BSA solution for 30 min at 45°C, washed twice with RNAse-free water for 2 min, rinsed with isopropanol and spin-dried for 3 min at 1500 g. Microarray hybridizations were performed overnight at 45°C, with continuous agitation, in a HS4800 Hybridization Station (Tecan Benelux BV). Finally, the arrays were washed automatically in the Hybridization Station using: 2x SSC/0.05% SDS (at 45°C), 1x SSC and 0.2x SSC (at room temperature), and dried under a stream of N2, before scanning.

### Data extraction and analysis

Arrays were scanned in a ScanArray Express HT scanner (Perkin Elmer, Nederland BV) and spot intensities were quantified using Imagene software (Bio Discovery Inc, El Sequndo, CA, USA). To balance Cy3 and Cy5 spot intensities, Loewess normalization per subarray was performed using limma-package (http://bioinf.wehi.edu.au/limma/) from Bioconductor (http://www.bioconductor.org) [23], [24]. To scale between arrays, the global median intensity per array was set at 1000. Dye intensities below 200 were then thresholded at 200, to minimize noise and make fold-change on the low-intensity range more robust against outliers. Spots with intensities below the threshold (200) for both Cy3 and Cy5 channels in more than 2 of the 4 arrays performed per subline were excluded from the analysis. Sample to reference ratios were then calculated and 2log transformed. Spots that showed opposite effects for the dye-swap/biological replicates were excluded from further analysis; effects were called opposite if the mean 2log ratio for the per subline were ≥0.5 for one dye and below ≤−0.5 for the dye-swap. Following normalization and all the above-mentioned quality controls, the 2log intensity ratios were averaged for the replicates of each subline. This data was stored in SRS7 (Sequence Retrieval System version 7, Lion Bioscience AG, Heidenberg, Germany), which was also used for the comparisons with other previously published/publicly available databases [25]. The microarray data was deposited in the Gene Expression Omnibus repository (http://www.ncbi.nlm.nih.gov/geo/ under the GEO accession number GSE21596). Hierarchical clustering and data visualization was performed using Cluster and TreeView programs (Eisen Labs: http://rama.lbl.gov). Significance Analysis of Microarrays (SAM; http://www-stat.stanford.edu/~tibs/SAM) was used to determine which genes were statistically different between stimulated samples and non-stimulated references. Gene ontology clustering was performed using Database for Annotation, Visualization and Integrated Discovery (DAVID: http://david.abcc.ncifcrf.gov) [26], [27]. The pathway and functional analyses were generated through the use of Ingenuity Pathways Analysis (Ingenuity® Systems, www.ingenuity.com).

### cDNA synthesis and RT- PCR analysis

Normal and tumor specimens from patients used for quantitative real-time RT-PCR analysis were obtained from the frozen tissue bank of the Erasmus Medical Center (Rotterdam, the Netherlands). The specimens were collected between 1984 and 2001. The experimental protocols were approved by the Erasmus MC Medical Ethics Committee according to the Medical Research Involving Human Subjects Act. Additional information about these specimens was provided previously.[28] Total RNA was isolated as described above and cDNA was synthesized using MMLV-reverse transcriptase kit and Oligo(dT)_12–18_ primer (Invitrogen), according to manufacturer's protocol. cDNA samples were stored at −20°C. TaqMan real-time PCR analysis was performed in an ABI Prism 7700 Sequence Detection System (Applied Biosystems, Foster City, CA), using AmpliTaq Gold DNA polymerase (Applied Biosystems), according to manufacturer's specifications. Validated primers and probes from TaqMan Gene Expression Assays (Applied Biosystems) were used for quantification of VAV3 (Hs00916821_m1), TWIST1 (Hs00361186_m1), DKK3 (Hs00951307_m1) and GAPDH (Hs99999905_m1), according to the PCR settings provided by Applied Biosystems. PBGD was quantified using 0.33 µM of primers forward: CATGTCTGGTAACGGCAATG and reverse: GTACGAGGCTTTCAATGTTG primers, in Power SybrGreen PCR Master Mix (Applied Biosystems), according to thermocycling protocol recommended by the manufacturer. Transcript quantities for each sample were normalized against the average of two endogenous references and relative to a calibrator. The two housekeeping genes used as endogenous references were *PBGD* and *GAPDH*; a mixture of cDNAs from prostate carcinoma xenografts was used as the calibrator. Graphs and statistics were performed with GraphPad Prism (version 3.0). P-values <0.05 were considered significant.

## Results

### Differential gene expression profile between the androgen-responsive PC346C and its therapy-resistant sublines

**Figure 1:**
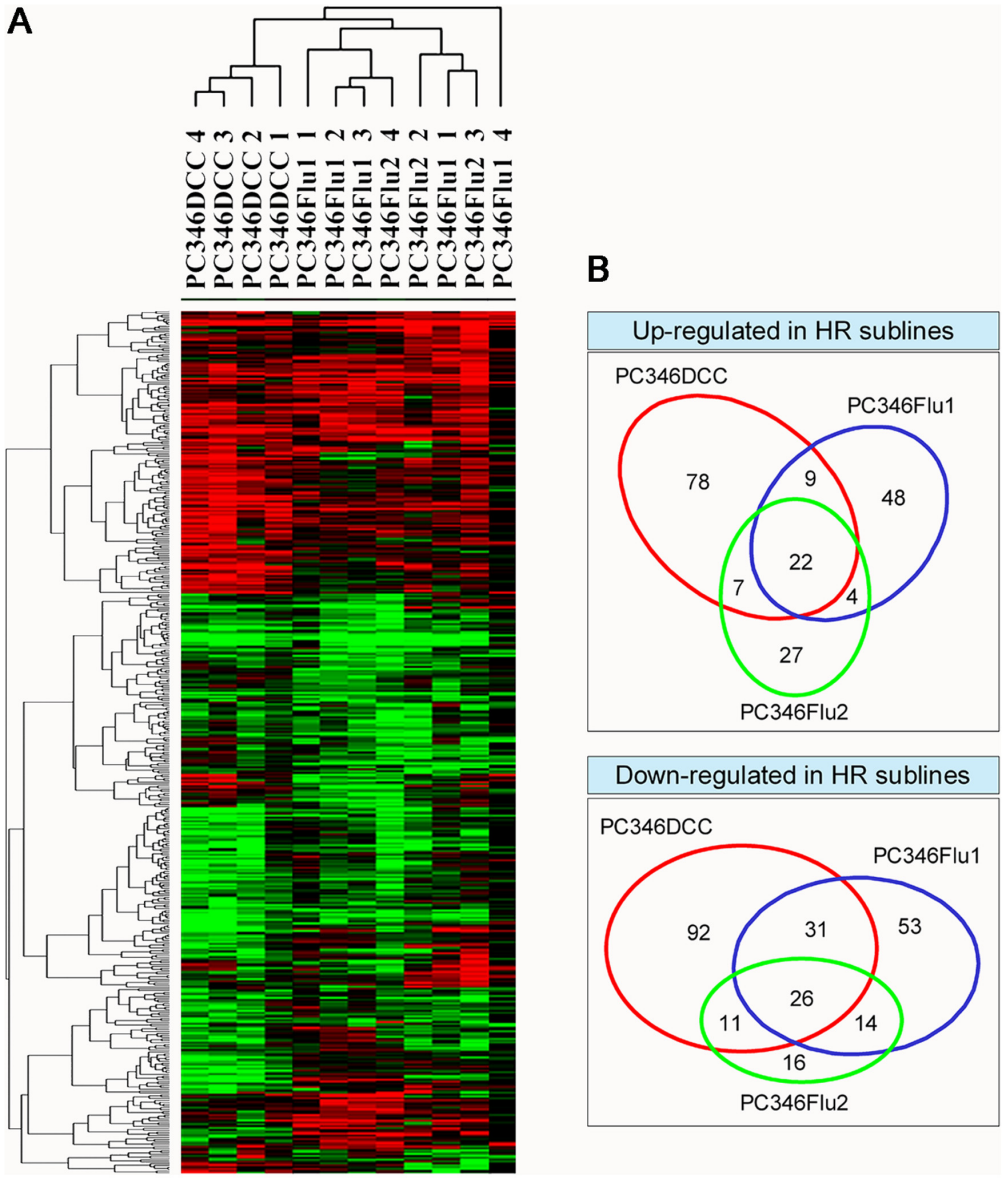
Differentially-expressed genes in PC346DCC, PC346Flu1 and PC346Flu2 sublines compared to the androgen-responsive PC346C. PC346C was cultured in complete medium with 0.1 nM R1881, whereas the hormone-refractory sublines were culture in dextran-coated charcoal stripped medium (PC346DCC), supplemented with 1 µM of the antiandrogen hydroxyflutamide (PC346Flu1 and PC346Flu2). A) Heat-map representation: red and green colors represent up-regulation and down-regulation, respectively, whereas black indicates no difference between sublines and parental PC234C cells. Grey squares indicate missing data, either due to low expression levels, poor data quality or absence of probes for the respective transcript in the array platform used for the study. B) Venn-diagram of the number of regulated genes in the different sublines.

Expression array analysis was performed to explore whether the AR pathway is still active in the hormone-therapy refractory cells under androgen-deprived conditions and identify putative alternative growth/survival pathways. Each of the therapy-resistant sublines were cultured in their respective selection medium (steroid-stripped medium for PC346DCC, supplemented with 1 µM OH-flutamide for PC346Flu1 and Flu2) and hybridized on the microarrays, together with the parental androgen-responsive PC346C (cultured in complete medium supplemented with 0.1 nM R1881). To account for the biological variability and dye-preferential binding to oligonucleotides on the microarray, four arrays were performed per condition, using two independent cell passages in dye-swap. Variation in expression pattern was analysed per therapy-resistant subline, and spots were considered to be differentially expressed if the absolute 2log ratio ≥ 0.5 (ratio ≥ 1.42 or ≤0.71) for at least three of the 4 arrays and for the average of all 4 arrays. According to these criteria, there were a total of 487 differentially regulated transcripts in the therapy-resistant sublines compared to androgen-sensitive PC346C, most of which were overlapping all three refractory sublines (Fig. 1). With 276 differentially regulated transcripts, PC346DCC showed the strongest divergence from the parental line, whereas PC346Flu2 revealed the least alterations (127 transcripts). Significance Analysis of Microarrays (SAM) was used to determine statistical significance of the selected genes and, at a 5% false discovery rate, 392 of the 487 (80%) selected reached statistical significance. The top 100 differentially expressed genes between the therapy-resistant and the androgen-responsive cell lines, respective expression ratios and statistical analysis are presented in Tables 1 and 2. A comprehensive list of all the regulated genes per subline is provided in Tables S1 to S3. Interestingly, a considerable proportion (64/487) of the differentially regulated genes clustered at distinct genomic locations on chromosomes 4, 5, 6, 8, 11 and 18 (p-value<0.05; Table 3).

**Table 1:** Top 50 genes overexpressed in the therapy-resistant cell lines, expression ratios and SAM q-values.

GenBank	Cytoband	HUGO symbol	HUGO Gene Name	PC346DCC		PC346Flu1		PC346Flu2	
				2log ratio	qvalue	2log ratio	qvalue	2log ratio	qvalue
NM_004982	12p11.23	KCNJ8	potassium inwardly-rectifying channel subfamily J member 8	2.7	0.000	1.2	0.000	1.9	0.000
NM_000790	7p11	DDC	dopa decarboxylase aromatic L-amino acid decarboxylase	1.9	0.005	0.1	0.253	2.4	0.218
NM_013452	Xp22	VCX	variable charge X-linked	2.3	0.000	1.6	0.015	1.7	0.049
NM_014269	4q34	ADAM29	ADAM metallopeptidase domain 29	1.5	0.000	1.4	0.011	2.2	0.000
NM_004750	19p12	CRLF1	cytokine receptor-like factor 1	2.1	0.000				
NM_006113	1p13.3	VAV3	vav 3 guanine nucleotide exchange factor	2.0	0.000	0.2	0.031	0.4	0.218
NM_003226	21q22.3	TFF3	trefoil factor 3 intestinal	1.9	0.000	−0.5	0.211	1.5	0.049
NM_001072	2q37	UGT1A1	UDP glucuronosyltransferase 1 family polypeptide A1	0.2	0.380	1.7	0.015	−0.1	0.714
NM_006332	19p13.1	IFI30	interferon gamma-inducible protein 30	1.7	0.000	1.1	0.025	0.7	0.218
NM_012463	12q24.31	ATP6V0A2	ATPase H transporting lysosomal V0 subunit a2	0.8	0.028	1.2	0.051	1.7	0.000
AB037810	1q42.2	SIPA1L2	signal-induced proliferation-associated 1 like 2	0.9	0.046	1.5	0.000	1.7	0.049
NM_001823	14q32	CKB	creatine kinase brain	1.7	0.000	1.5	0.015	1.6	0.000
NM_016084	17p11.2	RASD1	RAS dexamethasone-induced 1	1.6	0.000	1.2	0.000	0.5	0.218
NM_005794	14q11.2	DHRS2	dehydrogenase/reductase SDR family member 2	1.4	0.003	1.5	0.071	1.2	0.051
NM_013253	11p15.2	DKK3	dickkopf homolog 3 Xenopus laevis	−0.6	0.005	1.4	0.000	0.7	0.218
AK026892	22q13.31	CERK	ceramide kinase	1.4	0.000	0.0	0.321	0.5	0.218
NM_006721	10q22	ADK	adenosine kinase	1.4	0.000	0.1	0.422	0.7	0.194
NM_005804	19p13.12	DDX39	DEAD Asp-Glu-Ala-Asp box polypeptide 39	1.4	0.000	0.3	0.253	0.8	0.194
U58096	Yp11.2	TSPY1	testis specific protein Y-linked 1	1.0	0.037	1.4	0.000		
S67154	3q28	EIF4A2	eukaryotic translation initiation factor 4A isoform 2	0.1	0.589	1.3	0.021	−0.5	0.101
AL049949	10q22.3	C10orf56	chromosome 10 open reading frame 56	1.3	0.000	0.5	0.048	0.4	0.218
NM_016639	16p13.3	TNFRSF12A	tumor necrosis factor receptor superfamily member 12A	0.6	0.028	1.3	0.000	0.5	0.218
NM_001902	1p31.1	CTH	cystathionase cystathionine gamma-lyase	1.3	0.020	0.5	0.044	0.7	0.194
NM_003516	1q21.2	HIST2H2AA3	histone cluster 2 H2aa3	−0.1	0.645	1.2	0.000	0.8	0.194
L07383	3p21.3	RPSA	ribosomal protein SA	0.1	0.513	1.2	0.071		
Y09836		ORF*	3 UTR of hypothetical protein ORF1*	0.7	0.046	1.0	0.011	1.2	0.101
NM_003712	19p13	PPAP2C	phosphatidic acid phosphatase type 2C	1.2	0.000	0.9	0.044	1.2	0.000
NM_014214	18p11.2	IMPA2	inositol myo -1 or 4 -monophosphatase 2	0.3	0.242	0.0	0.515	1.1	0.000
Y11158	16p13.3	SNORA10	small nucleolar RNA H/ACA box 10	−0.1	0.513	1.1	0.000	0.5	0.218
NM_005311	7p12–p11.2	GRB10	growth factor receptor-bound protein 10	1.1	0.028	0.7	0.008	0.5	0.218
AF086251	11q21	SESN3	sestrin 3	0.1	0.534	1.1	0.048	1.1	0.049
NM_003234	3q29	TFRC	transferrin receptor p90 CD71	1.1	0.000	0.8	0.025	0.4	0.218
NM_014061	Xp11.22	MAGEH1	melanoma antigen family H 1	1.0	0.020	−0.5	0.090	0.0	0.725
NM_016192	2q32.3	TMEFF2	transmembrane protein with EGF-like and 2 follistatin-like domains 2	0.0	0.645	0.3	0.115	1.0	0.049
NM_003243	1p33–p32	TGFBR3	transforming growth factor beta receptor III	1.0	0.000	0.1	0.287	0.5	0.236
U90878	10q22-q26.3	PDLIM1	PDZ and LIM domain 1 elfin	1.0	0.000	0.4	0.135	0.4	0.287
AF190900	1q32.1	KLHL12	kelch-like 12 Drosophila	0.2	0.466	0.7	0.000	1.0	0.194
NM_002795	17q12	PSMB3	proteasome prosome macropain subunit beta type 3	0.5	0.113	0.6	0.063	1.0	0.000
NM_001814	11q14.2	CTSC	cathepsin C	1.0	0.000				
NM_000213	17q25	ITGB4	integrin beta 4	0.9	0.086	−1.3	0.000	0.1	0.777
AK021498	7q22.3	FLJ36031*	Hypothetical protein FLJ36031*	0.9	0.028	−0.1	0.321	0.1	0.755
NM_003524	6p21.3	HIST1H2BH	histone cluster 1 H2bh	0.2	0.380	0.9	0.000	0.2	0.384
NM_018303	6p25.3	EXOC2	exocyst complex component 2	0.9	0.000	0.2	0.253	0.2	0.636
NM_001262	1p32	CDKN2C	cyclin-dependent kinase inhibitor 2C p18 inhibits CDK4	0.5	0.211	0.1	0.459	0.9	0.194
NM_001831	8p21–p12	CLU	clusterin	0.1	0.534	0.9	0.000	0.7	0.049
NM_003311	11p15.5	PHLDA2	pleckstrin homology-like domain family A member 2	0.0	0.624	0.9	0.011	0.8	0.194
NM_004282	6p12.3-p11.2	BAG2	BCL2-associated athanogene 2	0.9	0.020	0.4	0.044	0.7	0.194
NM_001327	Xq28	CTAG1A	cancer/testis antigen 1A	0.6	0.046	0.9	0.000	0.6	0.194
AL157449	17q21.33	PPP1R9B	protein phosphatase 1 regulatory inhibitor subunit 9B	0.9	0.007	0.9	0.000	0.6	0.218
AF200348	2p25	PXDN	peroxidasin homolog Drosophila	−1.2	0.003	−1.3	0.000	0.9	0.064

*no approved HUGO symbol/name exists for this entry. If present, gene symbol/name from the UNIGENE database is given in alternative.

2log ratio>0 indicates overexpression in the therapy-refractory subline, compared to parental PC346C.

2log ratio<0 indicates down-regulation in the therapy-refractory subline.

**Table 2:** Top 50 genes down-regulated in the therapy-resistant cell lines, expression ratios and SAM q-values.

GenBank	Cytoband	HUGO symbol	HUGO GeneName	PC346DCC		PC346Flu1		PC346Flu2	
				2log ratio	qvalue	2log ratio	qvalue	2log ratio	qvalue
NM_017935	4q24	BANK1	B-cell scaffold protein with ankyrin repeats 1	−2.9	0.000	−1.8	0.000	−0.8	0.053
AF216077	9q32	COL27A1	collagen type XXVII alpha 1	−2.1	0.000	−2.8	0.000	−1.7	0.053
NM_014380	Xq22.2	NGFRAP1	nerve growth factor receptor TNFRSF16 associated protein 1	−0.2	0.513	−2.5	0.000	−2.7	0.000
AB042410	1p21.3	GPR88	G protein-coupled receptor 88	−2.4	0.000	−0.3	0.394	0.8	0.194
AK026813	7q21	STEAP2	six transmembrane epithelial antigen of the prostate 2	−2.3	0.000	−0.8	0.135	−0.8	0.176
AF188747	19q13.41	KLK2	kallikrein-related peptidase 2	−2.3	0.000	−0.7	0.063	−0.8	0.053
NM_012449	7q21	STEAP1	six transmembrane epithelial antigen of the prostate 1	−2.3	0.000	−0.9	0.036	−0.5	0.236
NM_003307	21q22.3	TRPM2	transient receptor potential cation channel subfamily M member 2	−2.1	0.000	0.0	0.536	−0.1	0.725
AB020968	6q22.2	MARCKS	myristoylated alanine-rich protein kinase C substrate	−1.6	0.000	−2.0	0.000	−1.3	0.053
M26663	19q13.41	KLK3	kallikrein-related peptidase 3	−2.0	0.009	0.0	0.207	−0.4	0.392
NM_004117	6p21.3-21.2	FKBP5	FK506 binding protein 5	−2.0	0.000	0.1	0.459	0.4	0.218
NM_001359	8q21.3	DECR1	2 4-dienoyl CoA reductase 1 mitochondrial	−1.9	0.000	−1.1	0.000	−1.6	0.053
NM_014333	11q23.2	CADM1	cell adhesion molecule 1	−1.0	0.000	−1.9	0.000	−0.1	0.725
AK026331	2q35	CHPF*	Chondroitin polymerizing factor*	−1.7	0.000	−0.8	0.048	−0.2	0.678
AB020637	11q21	ENDOD1	endonuclease domain containing 1	−1.7	0.000	−0.4	0.160	0.2	0.678
NM_012116	19q13.2	CBLC	Cas-Br-M murine ecotropic retroviral transforming sequence c	−1.6	0.000	−1.2	0.000	−0.4	0.218
X15667		GPRP*	Glutathione peroxidase-related protein GPRP*	−1.6	0.022	0.2	0.185	−0.8	0.053
NM_006167	8p21	NKX3-1	NK3 homeobox 1	−1.6	0.003	−0.1	0.459	−0.3	0.413
NM_006006	11q23.1	ZBTB16	zinc finger and BTB domain containing 16	−1.6	0.003	0.1	0.444	0.3	0.328
NM_003278	3p22-p21.3	CLEC3B	C-type lectin domain family 3 member B	−1.5	0.005	−1.3	0.000	−1.3	0.084
AL049963	4q22-q24	SLC39A8	solute carrier family 39 zinc transporter member 8	−1.5	0.000	−1.4	0.000	−0.9	0.053
AK000216	3p21.31	FLJ20209*	Hypothetical protein FLJ20209*	−1.5	0.000	−0.7	0.115	−0.7	0.084
AK000028	4q24	LOC90024*	Hypothetical LOC90024*	−1.5	0.000	−0.7	0.000	−0.5	0.120
NM_007011	15q26.1	ABHD2	abhydrolase domain containing 2	−1.5	0.000	−1.1	0.032	0.1	0.573
AK024917	1p22	DDAH1	dimethylarginine dimethylaminohydrolase 1	−0.3	0.380	−1.5	0.000	0.1	0.760
AF252283	13q21	KLHL1	kelch-like 1 Drosophila	−1.5	0.000	0.5	0.115	0.1	0.738
X75684	3q21-q25	TM4SF1	transmembrane 4 L six family member 1	0.6	0.211	−1.5	0.000	0.5	0.194
AL122055	6q21	CDC2L6	cell division cycle 2-like 6 CDK8-like	−1.4	0.000	−1.3	0.000	−1.2	0.000
NM_000944	4q21-q24	PPP3CA	protein phosphatase 3 formerly 2B catalytic subunit alpha isoform	−1.4	0.028	−1.2	0.000	−1.0	0.084
NM_016598	3p21.31	ZDHHC3	zinc finger DHHC-type containing 3	−1.4	0.003	−1.1	0.000	−0.9	0.053
NM_002151	19q11-q13.2	HPN	hepsin transmembrane protease serine 1	−1.0	0.000	−0.9	0.000	−1.4	0.053
NM_001311	14q32.33	CRIP1	cysteine-rich protein 1 intestinal	−1.4	0.018	0.4	0.101	−0.9	0.115
AL050367	10p13	C10orf38	chromosome 10 open reading frame 38	0.1	0.380	−1.4	0.000	−0.6	0.084
NM_005864	14q11.2-q12	EFS	embryonal Fyn-associated substrate	−1.4	0.009	0.4	0.135	−0.6	0.064
NM_005510	6p21.3	DOM3Z	dom-3 homolog Z C. elegans	0.5	0.211	0.1	0.422	−1.4	0.053
NM_019005	7p22-p21	FLJ20323*	Hypothetical protein FLJ20323*	−0.9	0.000	−1.3	0.000	−0.6	0.134
AK026517	11p12	EHF	ets homologous factor	−0.7	0.028	−1.3	0.000	−0.6	0.309
AF271070	12q13.11	SLC38A1	solute carrier family 38 member 1	−1.3	0.000	−0.7	0.044	−0.2	0.678
AF200348	2p25	PXDN	peroxidasin homolog Drosophila	−1.2	0.003	−1.3	0.000	0.9	0.064
NM_003255	17q25	TIMP2	TIMP metallopeptidase inhibitor 2	0.6	0.028	−1.3	0.031	−0.2	0.678
L29496	Xq11.2-q12	AR	androgen receptor	−1.3	0.018	0.9	0.000	−0.3	0.194
NM_000213	17q25	ITGB4	integrin beta 4	0.9	0.086	−1.3	0.000	0.1	0.777
D80010	2p25.1	LPIN1	lipin 1	−1.2	0.000	−1.1	0.015	−0.9	0.053
NM_007173	11q14.1	PRSS23	protease serine 23	−1.2	0.018	−0.9	0.021	−0.9	0.101
NM_002165	20q11	ID1	inhibitor of DNA binding 1 dominant negative helix-loop-helix protein	−1.2	0.018			−0.6	0.500
NM_017860	1q21.2	C1orf56	chromosome 1 open reading frame 56	−0.5	0.037	−1.2	0.000	−0.6	0.053
NM_004457	2q34-q35	ACSL3	acyl-CoA synthetase long-chain family member 3	−1.2	0.009	−0.7	0.025	−0.3	0.101
NM_005045	7q22	RELN	reelin	−1.2	0.003	−0.6	0.101	−0.2	0.714
NM_005544	2q36	IRS1	insulin receptor substrate 1	−1.2	0.020	−0.1	0.394	0.0	0.714
AK024495	11p15.5	LRRC56	leucine rich repeat containing 56	−1.2	0.000	0.2	0.321	0.5	0.218

*no approved HUGO symbol/name exists for this entry. If present, gene symbol/name from the UNIGENE database is given in alternative.

2log ratio>0 indicates overexpression in the therapy-refractory subline, compared to parental PC346C.

2log ratio<0 indicates down-regulation in the therapy-refractory subline.

**Table 3:** Chromosomal clustering of the differentially expressed transcripts.

Cell line	#genes regulated	up/down regulated	Cytoband	#genes cytoband*	Bonferroni p-value
PC346DCC	7	down	4q21-24	56	0.003
PC346DCC	9	down	5q11-23	130	0.028
PC346DCC	6	down	6q14-23	65	0.026
PC346DCC	8	down	8p11-22	74	0.003
PC346DCC	10	down	8q11-24	131	0.006
PC346Flu1	6	down	11p11-15	92	0.068
PC346Flu2	14	up	18	109	<0.0001

p-values determined by Fisher's exact test with Bonferroni correction for multiple testing.

*number of genes located in the cytoband that are expressed by the indicated cell line.

### AR pathway is down-regulated in PC346DCC

**Figure 2:**
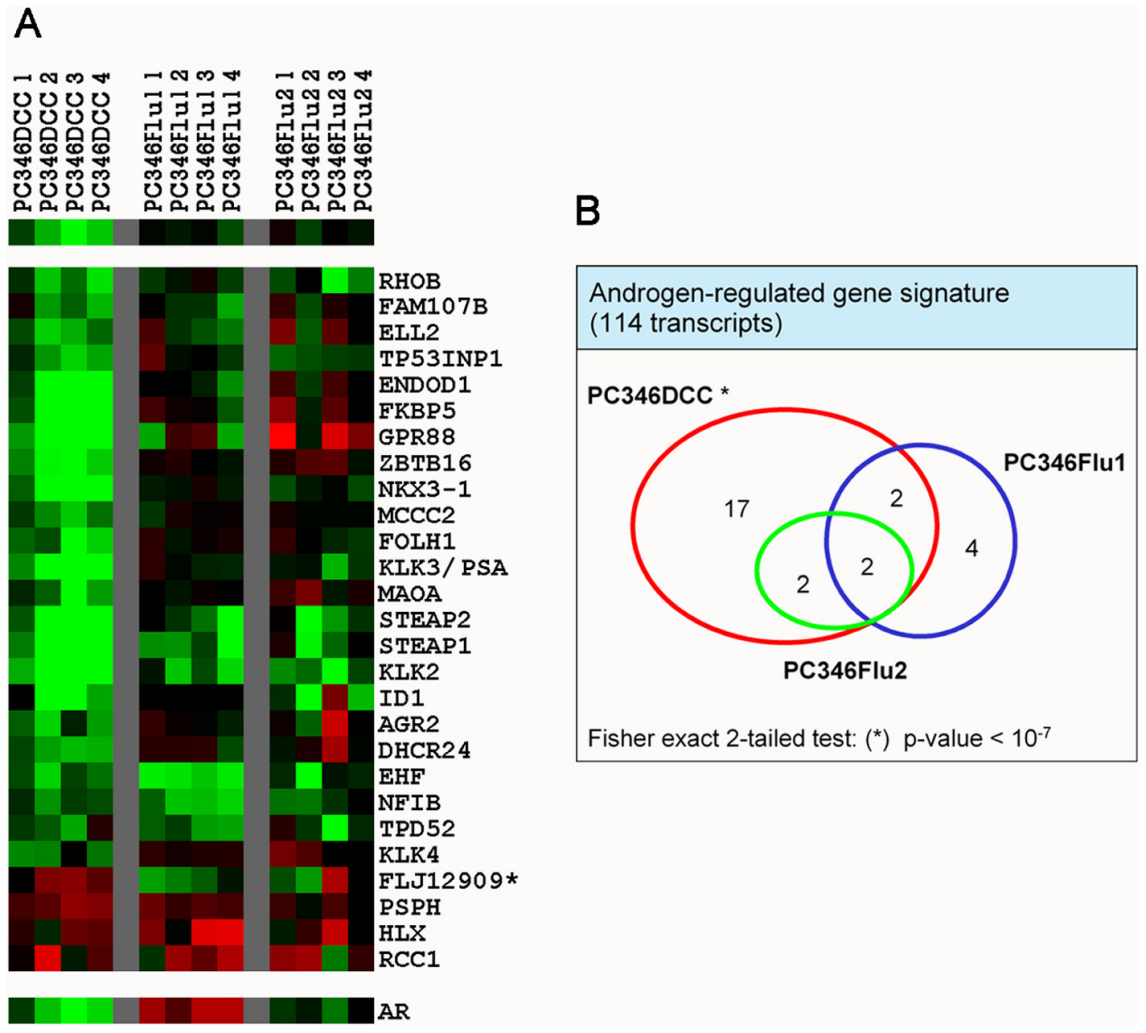
Activation state of the AR pathway in the PC346DCC, PC346Flu1 and PC346Flu2. Differentially-expressed genes in PC346 hormone-refractory sublines versus parental PC346C were linked to a previously established androgen-response gene signature (see Materials and Methods section). (A) Heat-map representation of androgen-responsive genes deregulated in any of the PC346 hormone-refractory sublines. Color scheme as described in Fig. 1. (B) Venn-diagram and respective statistics.

To investigate the activation state of the AR pathway in the PC346DCC, PC346Flu1 and PC346Flu2, the SRS database was used to link and compare our present data with a previously established androgen-response gene signature (Fig. 2A). This androgen-response signature was determined by expression microarray analysis, after stimulation of the different PC346 cell lines with the synthetic androgen R1881 or the antiandrogen hydroxyflutamide (Table S4). Of the 487 differentially-regulated transcripts in the therapy-resistant sublines, only 27 were AR target genes (<6%), indicating that other genes and pathways are also involved in hormone-therapy refractory proliferation of these sublines. Furthermore, AR target genes were down-regulated in PC346DCC (p-value  = 10^−7^), whereas their expression in PC346Flu1 and PC346Flu2 was not significantly affected (Fig. 2B). However, although not statistically significant for the AR pathway as a whole, PC346Flu1 and PC346Flu2 did show lower induction of some androgen-responsive genes such as KLK2, STEAP1, STEAP2 and EHF.

### Gene ontology and pathway analysis identifies cancer signature

**Figure 3:**
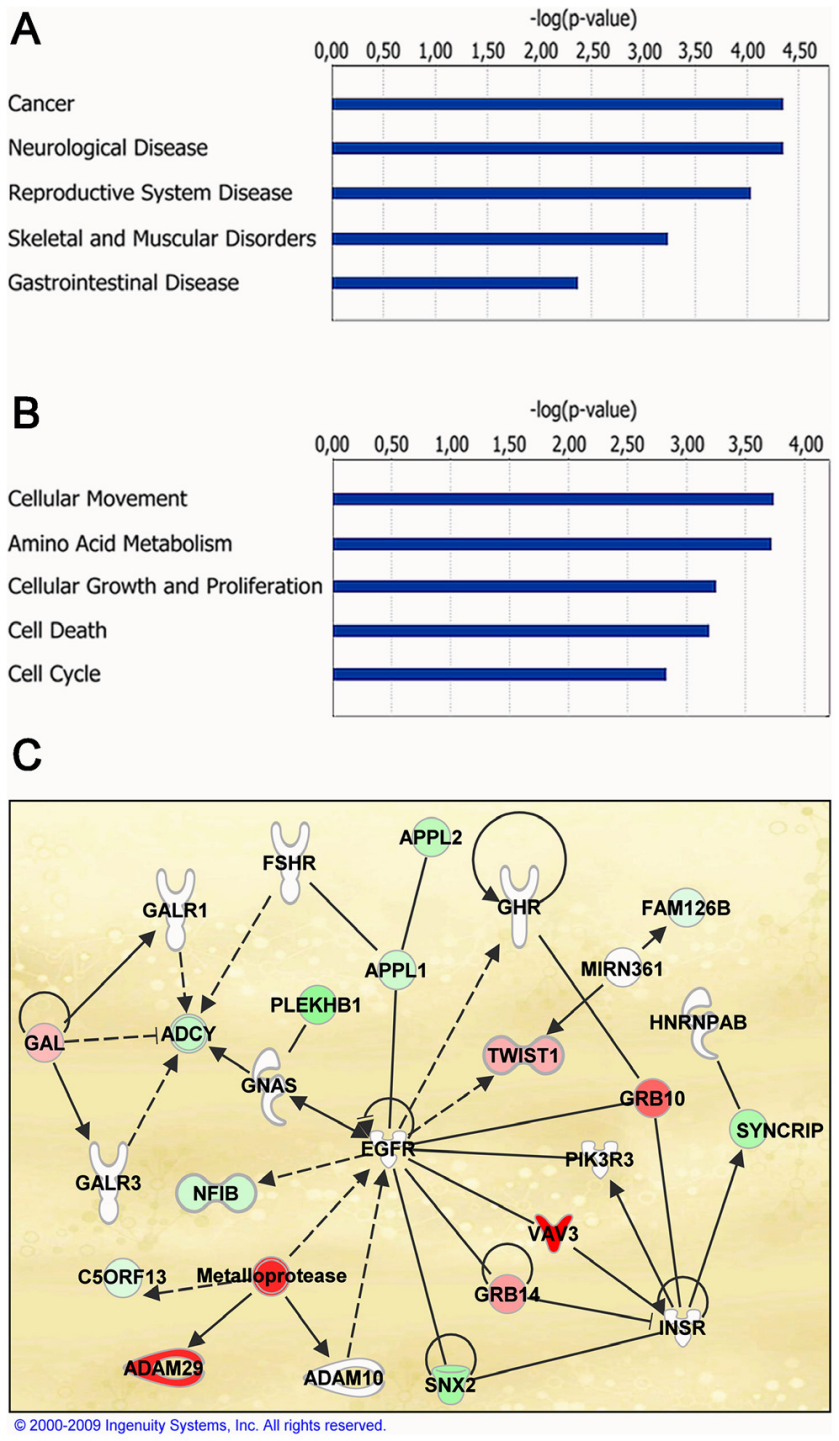
Biological processes deregulated in the hormone-refractory sublines. Top 5 biological functions enriched in the therapy-resistant sublines: (A) diseases and disorders, (B) molecular and cellular functions. (C) Example of Network analysis for PC346DCC showing deregulation of hormone and growth-factor receptor signaling: up-regulated genes are represented in red and repressed genes in green. Analysis was performed using Ingenuity Pathway Analysis software (www.ingenuity.com).

The selected 487-gene signature was classified according to Gene Ontology (GO) Biological Processes using the Database for Annotation, Visualization and Integrated Discovery (DAVID) [26], [27]. Annotation clustering analysis showed enrichment in categories involved in organ development, reproductive system differentiation, cellular growth, differentiation and apoptosis (Table 4). Ingenuity Pathway Analysis was used to identify enrichment in “diseases and disorders”, “molecular and cellular functions”, and to search for intrinsic pathways/networks within the selected gene sets (www.ingenuity.com). Cancer and reproductive system disease were ranked in the top 3 of “diseases and disorders”, which logically confirmed the enrichment of genes associated with PCa, such as hepsin, clusterin, vitamin D receptor, trefoil factor 3, tumor protein D52, the AR itself and several of its target genes (Fig. 3A and 3B, respectively). Furthermore, we used Network analysis to screen the 276-gene signature of PC346DCC for potential alternative growth pathways that could be involved in bypassing the AR signaling. Interestingly, signaling via growth-hormone receptor (GHR), insulin receptor (INSR) and epidermal growth factor receptor was among the top 10 Networks (score  = 20) showing deregulation in PC346DCC (Fig. 3C).

**Table 4:** Biological processes significantly enriched in the therapy-resistant gene signature.

**Annotation Cluster 1**	**Enrichment Score: 4.42**	**count**	**P value**
	multicellular organismal development	86	1.8E-05
	anatomical structure development	78	7.7E-05
	system development	68	3.9E-05
**Annotation Cluster 2**	**Enrichment Score: 4.24**	**count**	**P value**
	cell differentiation	69	7.7E-05
	cell development	53	3.1E-05
**Annotation Cluster 3**	**Enrichment Score: 4.09**	**count**	**P value**
	apoptosis	38	4.1E-05
	programmed cell death	38	5.0E-05
**Annotation Cluster 4**	**Enrichment Score: 2.68**	**count**	**P value**
	regulation of phosphorylation	8	1.7E-03
	regulation of phosphate metabolic process	8	2.3E-03
**Annotation Cluster 5**	**Enrichment Score: 2.67**	**count**	**P value**
	amino acid and derivative metabolic process	21	5.8E-04
	amine metabolic process	21	6.0E-03
	nitrogen compound metabolic process	22	6.7E-03
**Annotation Cluster 6**	**Enrichment Score: 2.15**	**count**	**P value**
	regulation of biological process	134	3.0E-03
	regulation of cellular process	124	1.4E-02
	regulation of gene expression	63	6.7E-02
**Annotation Cluster 7**	**Enrichment Score: 2.15**	**count**	**P value**
	neurogenesis	16	7.0E-03
	neuron differentiation	14	6.2E-03
**Annotation Cluster 8**	**Enrichment Score: 2.03**	**count**	**P value**
	cellular lipid metabolic process	29	3.8E-03
	lipid metabolic process	28	1.6E-03
**Annotation Cluster 9**	**Enrichment Score: 1.82**	**count**	**P value**
	DNA packaging	16	8.9E-03
	establishment and/or maintenance of chromatin architecture	15	1.7E-02
	chromosome organization and biogenesis	17	2.3E-02
	chromatin assembly or disassembly	9	2.1E-02
	nucleosome assembly	7	1.8E-02
**Annotation Cluster 10**	**Enrichment Score: 1.70**	**count**	**P value**
	development of primary sexual characteristics	7	2.0E-02
	sex differentiation	6	1.8E-02
	reproductive developmental process	6	2.2E-02
	gonad development	5	4.9E-02
**Annotation Cluster 11**	**Enrichment Score: 1.42**	**count**	**P value**
	growth	12	8.3E-02
	regulation of cell size	11	1.8E-02
	cell growth	10	3.7E-02

### Integrative analysis reveals genes deregulated in prostate cancer progression

**Figure 4:**
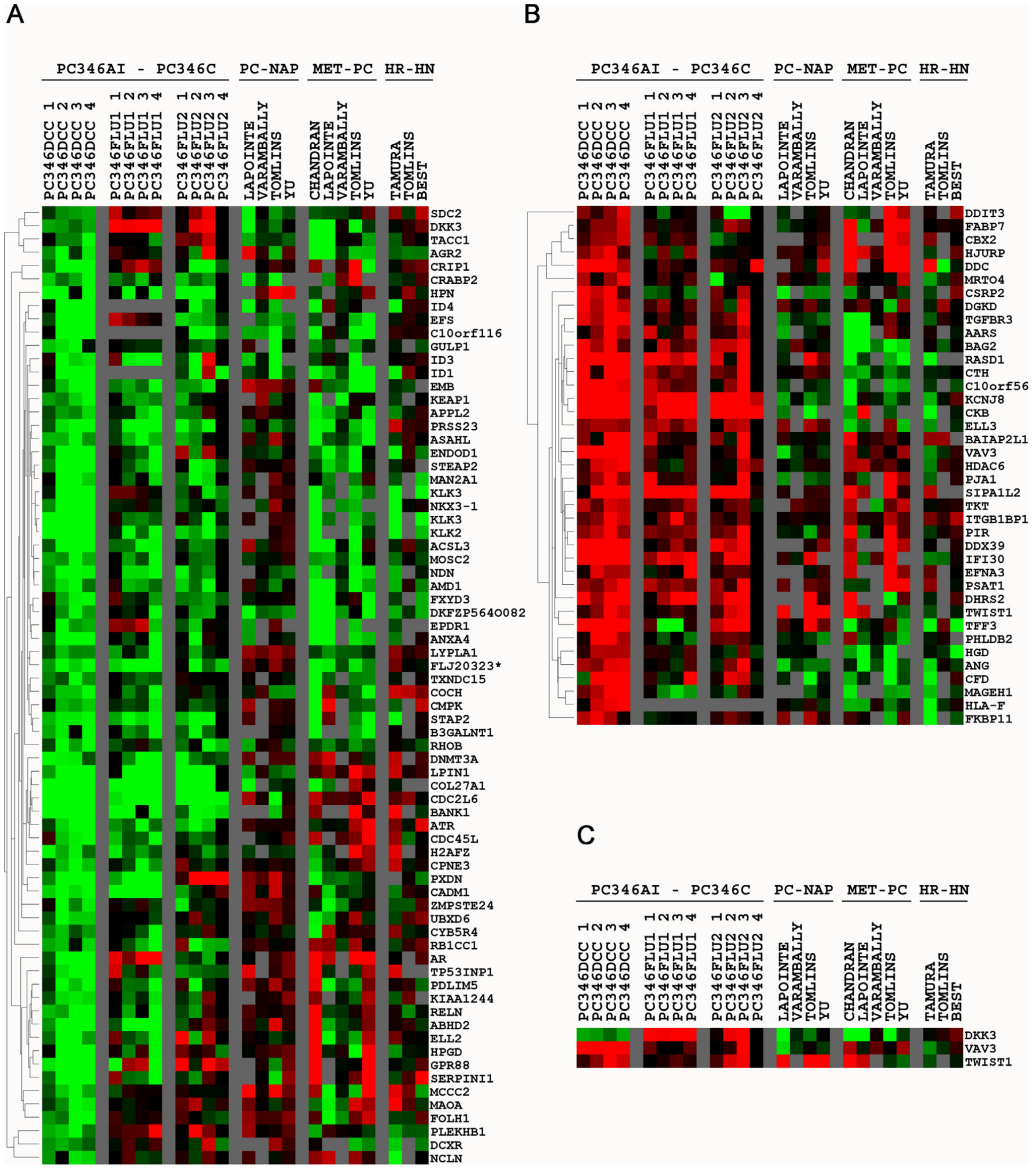
Expression of the androgen-independent PC346DCC signature genes in prostate cancer samples from patient tumors. The 276-gene signature from PC346DCC was linked to data from 7 prostate cancer microarray databases of primary (Lapointe, Varambally, Tomlins, Yu), metastatic (Chandran, Lapointe, Varambally, Tomlins, Yu) and hormone-therapy refractory tumors (Tamura, Tomlins and Best). Only genes present in at least 5/7 databases (209 genes) and deregulated in at least 3/7 (111 genes) were included in the analysis. Heat-map representation of (A) 72 overexpressed and (B) 39 repressed genes in PC346DCC. (C) Deregulated genes selected for further qPCR analysis. Color scheme as described in Fig. 1. Grey squares indicate missing data, either due to low expression levels, poor data quality or absence of probes for the respective transcript in the array platform used for the study. PC-NAP: prostate cancer minus normal adjacent prostate; MET-PC: metastasis minus primary prostate tumors; HR-HN: hormone-therapy refractory minus hormone-naïve tumors.

To identify genes modulated in PCa that could explain hormone-therapy refractory growth through bypass of the AR pathway, we linked the 276-gene signature from PC346DCC with data from seven PCa microarray studies published previously (Table 5) [14], [29], [30], [31], [32], [33], [34]. Only genes present in at least 5/7 databases (209 genes) and deregulated in at least 3/7 (111 genes) were included for further analysis. Hierarchical clustering performed on the signature genes (first column), next to primary cancer vs. normal prostate (second column), metastatic cancer vs. primary cancer (third column), and finally hormone-refractory vs. hormone-naïve disease (fourth column), is shown in Fig. 4. Approximately 30% of the genes differentially expressed in PC346DCC were found to be consistently deregulated in metastatic PCa compared to organ-confined disease.

**Table 5:** Description of prostate cancer databases linked via SRS.

First Author	Reference samples	Test samples
Best (2005)^29^	10 hormone-naive prostate cancers	10 hormone-refractory primary prostate tumors
Chandran (2007)^30^	64 primary prostate tumor samples	24 hormone-refractory metastatic samples (4 patients)
Lapointe (2004)^31^	41 benign prostate tissue adjacent to cancer	62 primary prostate tumor samples
		9 lymph node metastasis
Tamura (2007)^32^	10 hormone-naive prostate cancers	18 hormone-refractory primary and metastatic tumors
Tomlins (2007)^14^	15 benign epithelial tissue adjacent to cancer	30 primary prostate tumor samples
		3 hormone-naive and 17 hormone-refractory metastasis
Varambally (2005)^33^	5 benign prostate tissues	5 clinically localized prostate cancers
Yu (2004)^34^	60 benign prostate tissue adjacent to cancer	5 hormone-refractory metastatic samples
	23 disease free donor prostate tissue	62 primary prostate tumors
		24 hormone-refractory metastasis

### TWIST1, DKK3 and VAV3 as markers for disease diagnosis and prognosis

Based on their recognized pathological functions and consistent deregulation in multiple PCa databases, twist homolog 1 (TWIST1), vav 3 guanine nucleotide exchange factor (VAV3) and dickkopf homolog 3 (DKK3) were selected for their potential role in the bypass of the AR pathway. In this manner, TWIST1 and VAV3 are putative oncogenes involved in growth hormone signaling, as revealed by Ingenuity Pathway Analysis (Fig. 3C). On the other hand, DKK3 is a tumor suppressor, showing strong down-regulation in the datasets from Chandran *et al.*, Lapointe *et al.* and Varambally *et al.* (Fig. 4C). Whereas TWIST1 showed consistent up-regulation in primary and metastatic PCa datasets from Varambally *et al.*, Yu *et al.*, Lapointe *et al.* and Chandran *et al.*, VAV3 was down-regulated in primary tumors followed by up-regulation in metastasis (Fig. 4C).

**Figure 5:**
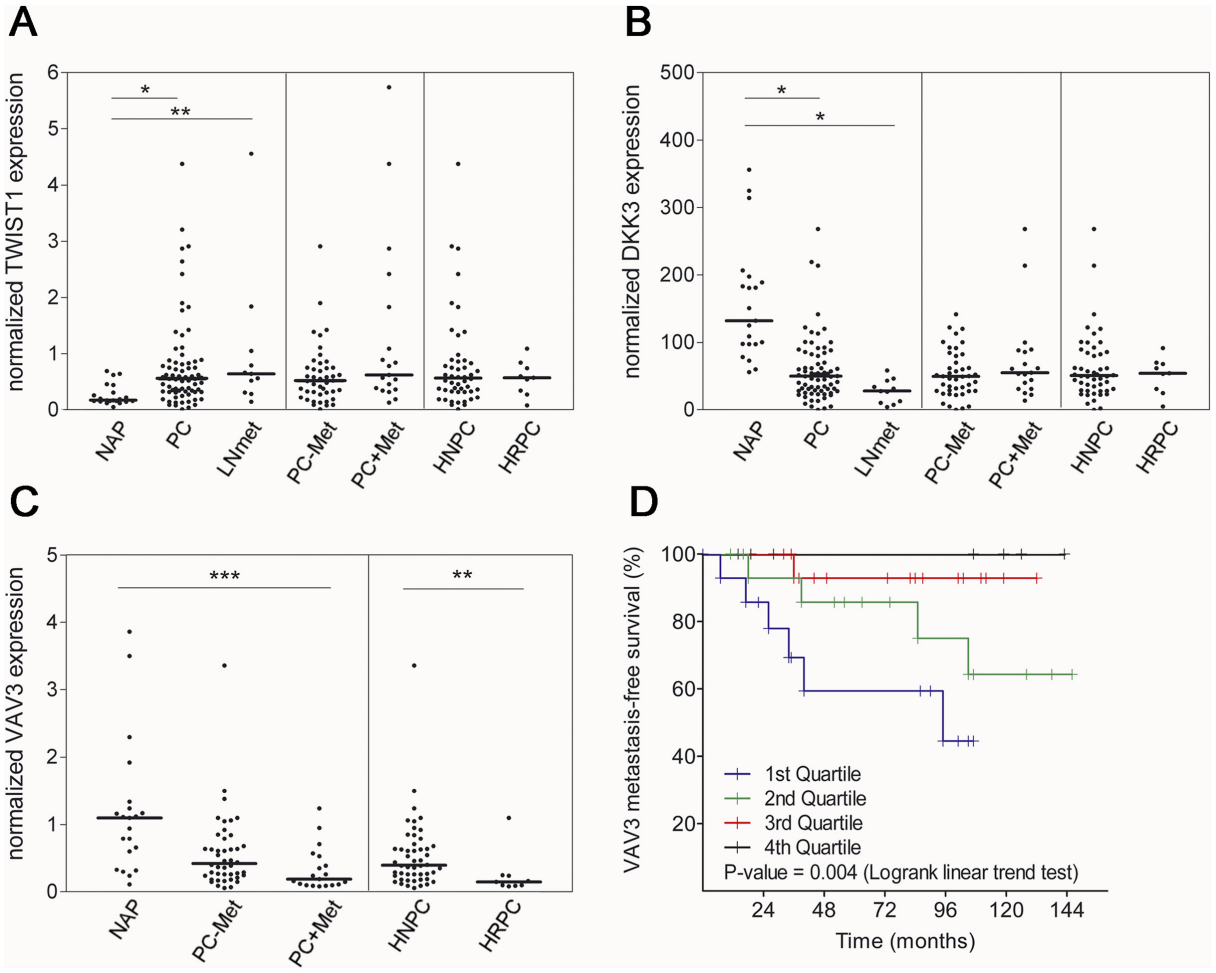
Quantitative RT-PCR analysis of TWIST1, DKK3 and VAV3 in an independent set of prostate samples. Prostate tumor samples were obtained by radical prostatectomy or transurethral resection of the prostate of patients being operated at Erasmus MC clinic. This panel contains 21 benign prostate tissue samples and 74 adenocarcinomas at different disease stages. (A) TWIST1; (B) DKK3; (C) VAV3 expression in prostate samples; (D) VAV3 metastasis-free survival analysis. NAP: normal adjacent prostate; PC: primary prostate cancer; LNmet: lymph node metastasis; PC-Met: non-progressive organ-confine prostate cancer; PC+Met: primary tumor from progressive prostate cancer that either had or developed metastasis during subsequent follow-up; HN: hormone-naïve; HR: hormone-therapy refractory; (*) p-value ≤0.0001 and (**) p-value ≤0.005 using Mann-Whitney two-tailed test. (***) p-value ≤0.0001 with Post linear-trend test.

Quantitative RT-PCR was performed on an independent set of prostate samples, obtained by radical prostatectomy or transurethral resection of the prostate of patients being operated at Erasmus MC clinic. This panel contained 21 benign prostate tissue samples and 74 adenocarcinomas at different disease stages. Quantitative PCR analysis showed up-regulation of TWIST1 in primary PCa samples and lymph node metastasis (P-value  = 0.0001 and 0.002, respectively). No difference was observed between hormone-refractory (HRPC) and hormone-naïve tumors (HNPC) (Fig. 5A). DKK3 expression was significantly decreased in PCa and lymph node metastasis (P-value ≤0.0001), although no difference was observed during progression from organ-confined to metastatic or hormone-refractory disease (Fig. 5B). VAV3 expression decreased gradually during PCa progression, with the lowest levels observed in metastatic prostate tumors (P-value  = 0.0001 for Post linear-trend test) and hormone-refractory samples (P-value  = 0.005 for HNPC vs. HRPC; Fig. 5C). Lymph node samples were removed from the VAV3 analysis in Fig. 5C, because VAV3 was highly expressed in normal lymph node compared to normal prostate tissues (data not shown). In these settings, the presence of remnants of normal lymph node tissue can lead to over-estimation of the real VAV3 quantity in lymph node metastasis. Kaplan-Meier analysis showed a direct correlation between VAV3 expression and metastasis-free survival (P-value  = 0.004 for Logrank trend test; Fig. 5D).

## Discussion

In the present study we used microarray analysis to identify differences in the gene expression pattern of the androgen-responsive PC346C cell line and its therapy-resistant sublines: PC346DCC, PC346Flu1 and PC346Flu2. This analysis detected 487 transcripts differentially regulated in the hormone-therapy refractory cells versus the parental PC346C. Many of these were common to all therapy-resistant sublines, despite the different AR pathway modifications, suggesting similar growth enhancing adaptations (Fig. 1). These shared genes could be divided in four main categories: regulation of cell cycle progression and proliferation (ex. HPN, NDN, ATR, ABL2, DHRS2), development and cellular differentiation (CLEC3B, KCNJ8, ADAM29, DNMT3A), fatty acid and steroid metabolism (LPIN1, DECR1, ACSL3) and intracellular signaling transduction (PPAP2C, PPP3CA, PRSS23, GRB10, SIPA1L2). Interestingly, 40 of the genes down-regulated in PC346DCC clustered in just four genomic locations: 4q21-24 (7 genes), 5q11-23 (9 genes), 6q14-23 (6 genes) and chr8 (18 genes). This is more than would be expected by chance. Also 6 genes down-regulated in PC346Flu1 and 14 up-regulated genes in PC346Flu2 clustered at 11q11-15 and chr18, respectively (Table 3). Duplication of chromosome 18 and loss of 8p has been previously reported in PC346Flu2 and PC346DCC, respectively, and may explain the clustering observed at these loci [20]. However, no evidence of chromosomal amplifications or deletions was detected at the other loci mentioned. A possible explanation is that epigenetic mechanisms, such as promotor methylation or histone modifications, may be involved in the transcriptional regulation of these large chromosomal regions. Indeed, expression of DNA methyltransferase DNMT3A and histone deacetylase HDAC6 was altered in PC346DCC, supporting this hypothesis. Nevertheless, losses at 4q, 5q, 6q, 8p and 11p have been frequently reported in PCa specimens and these loci are suspected of harbouring potential tumor suppressor genes [35], [36].

Previously, we have shown that PC346DCC, PC346Flu1 and PC346Flu2 display different AR modifications that resulted in distinct mechanisms of hormone-therapy refractory growth [20]. PC346DCC revealed very low levels of the AR and its target gene prostate specific antigen (PSA), and was insensitive to androgen stimulation in growth assays, promotor transactivation assays and expression microarray profiling (unpublished data) [20]. These results suggest that the AR pathway has been bypassed and is not essential for the growth of PC346DCC cells. The present study further substantiates this hypothesis by showing a strong down-regulation of AR target genes compared to the parental PC346C (Fig. 2). PC346Flu1, on the other hand, expresses high levels of the AR and previously showed a “super-activation” of this receptor in response to androgens, both in transactivation assays as in expression microarray analysis (unpublished data) [20]. Interestingly, the proliferation of this subline is inhibited by physiological concentration of androgens, and is optimal in the absence of this ligand. A possible explanation for this growth suppressive effect is that the “super-activation” of the AR by androgens in PC346Flu1 may be tilting the balance towards cellular differentiation [37], [38]. It is worth noting how few AR target genes are deregulated in PC346Flu1 versus the parental PC346C (Fig. 2). This suggests that the AR pathway remained active in the PC346Flu1 cells cultured in androgen-depleted medium supplemented with AR antagonist hydroxyflutamide. The few AR target genes that were differentially expressed in PC346Flu1 include EHF, NFIB and HLX, which are involved in development and differentiation processes. The third therapy-resistant subline studied here, PC346Flu2, carries the T877A AR mutation, well known for causing broadened receptor activation by non-androgenic ligands, including flutamide [39]. Consistent with the presence of this “promiscuous” AR, the growth of PC346Flu2, as well as expression of AR target genes, are stimulated by both the synthetic androgen R1881 and the antiandrogen hydroxyflutamide (unpublished data) [20]. In PC346Flu2, the presence of the T877A AR mutation allows for the maintenance of AR activity in the selection medium supplemented with 1 µM hydroxyflutamide. This is further substantiated in the present study by the observation that PC346Flu2 is the least divergent of the therapy-resistant sublines, with no more than 127 differentially regulated transcripts compared to the parental PC346C (Fig. 1).

Of the 487 transcripts differentially expressed between androgen-responsive PC346C cells and the therapy-resistant sublines only a few (∼5%) were AR-regulated genes. These results indicate that the gene expression differences observed are not largely explained by R1881 activating the AR transcriptional program in PC346C cells. Expression of AR-regulated cell cycle genes, such as A-, B- and D-type cyclins, cyclin-dependent kinases (CDK1, CDK2, CDK4, CDK6) and cyclin-dependent kinase inhibitors (CDKN1B, CDKN2A, CDKN2B), was generally unaffected [40], [41], [42], [43], [44], [45]. Cyclin D1 (CCND1) was the only cyclin found to be deregulated, with a 2-fold lower expression level in all three therapy-resistant sublines. However, apart from its role in cell cycle transitions, CCND1 is also a potent inhibitor of AR activity [46]. Since CCND1 expression is induced by androgens, higher expression in PC346C may reflect the R1881 supplementation in this cell line [47]. On the other hand, lower expression of CCND1 in the therapy-resistant sublines, in particular PC346Flu1 and PC346Flu2, may contribute to the maintenance of AR activity under selection conditions. Finally, one must keep in mind that cyclins and other cell cycle genes are mainly regulated during cell division through post-transcriptional mechanisms, including protein degradation, localization and phosphorylation [44], [48].

Both PC346Flu1 and PC346Flu2 sublines have an active AR and acquired hormone-therapy refractory proliferation through adaptations of the AR pathway. Therefore, to study alternative survival and growth pathways independent of the AR we further focused on the expression profile of PC346DCC cells. The Ingenuity Pathway Analysis program was used to screen the 276-gene signature of PC346DCC for potential gene Networks that could be involved in bypassing the AR signaling. Among the top 10 Networks, the signaling pathway via growth-hormone receptor (GHR), insulin receptor (INSR) and epidermal growth factor receptor (EGFR) as a potential candidate emerged (Fig. 3C). These receptors are not themselves deregulated in PC346DCC but several partners of this signaling pathway were, including VAV3 and TWIST1. VAV3 and TWIST1 are potential oncogenes, and while VAV3 can interact with and cross-activate signaling via hormone and growth receptors, expression of TWIST1 is in turn stimulated by EGF and IGF1 [49], [50], [51], [52]. The effect of peptide growth factors on androgen independent proliferation of prostate cancer cells is mainly mediated via the Ras/MAPK, PI3K/AKT and STAT3 signaling cascades [53], [54]. These signaling pathways may directly activate transcription of genes involved in cell survival, proliferation and migration, but may also indirectly activate the AR pathway in a ligand-independent manner. In the absence of androgens, MAPK and AKT kinases may induce AR phosphorylation and activation, whereas STAT3 can bind ligand-free AR and facilitate its translocation to the nucleus. Thus peptide growth factors may function as alternative survival/growth pathways, for example in the subgroup of AR negative prostate tumors, and/or as an adaptation of AR pathway by preserving AR activity under androgen ablation conditions. Recent reports have implicated Src, a member of the Src-family kinases, in the proliferation of hormone-refractory tumors. Src kinase was found to be overexpressed in prostate cancer, where Src inhibitors decreased proliferation and invasion of cell lines and xenografts [55], [56]. Src-family kinases are nonreceptor protein tyrosine kinases responsible for signal transduction in many cellular and oncogenic processes. Src kinases are activated upon binding to cell surface receptors, such as G-protein coupled receptors, growth factor receptors and integrins, or other intracellular protein kinases [55], [57]. In turn, activated Src kinases signal via the MAPK, PI3K, STAT3 and FAK pathways. Src kinases are also activators of VAV3 and TWIST1, either directly by phosphorylating and releasing inhibitory tyrosine Y173 of VAV3, or indirectly by activating STAT3, a transcription activator for TWIST1 [58], [59]. These results link the MAPK, PI3K, STAT3 and Src kinase signaling to growth factor pathways and to our candidate genes selected for hormone-refractory progression. Both VAV3 and TWIST1 are overexpressed in PC346DCC cells, and survey of 7 previous microarray studies revealed a consistent up-regulation in metastatic patient material (Fig. 4C). Another interesting candidate gene for the bypass of the AR pathway was the DKK3 tumor suppressor, which was down-regulated in PC346DCC and multiple databases of primary and metastatic tumors (Fig. 4C). While compelling evidence links these genes to PCa pathogenesis, it is not known whether TWIST1, VAV3 or the tumor suppressor DKK3 may have a functional role in developing resistance to hormonal therapy.

TWIST1 is a helix-loop-helix transcription factor, regulator of embryonic morphogenesis, and highly expressed in many types of human cancer [60]. The role of TWIST1 as a potential oncogene was first suggested through a functional screen for cDNAs that could counteract the pro-apoptotic effects of the MYC oncogene. In that study, TWIST1 expression bypassed P53-induced growth arrest and promoted colony formation, consistent with a potential role as oncoproteins [61]. Yang *et al.* showed that suppression of TWIST1 expression in highly metastatic mammary carcinoma cells specifically inhibited their ability to metastasise to the lung, while ectopic expression resulted in activation of mesenchymal markers and induction of cell motility [62]. Previous studies also implicated TWIST1 in the development and progression of PCa, showing that its expression was up-regulated in prostate adenocarcinomas and correlated with Gleason grading and increased metastatic potential [63], [64]. Furthermore, inactivation of TWIST1, through small interfering RNA, induced growth arrest and suppressed migration and invasion abilities in androgen-independent PCa cell lines DU145 and PC3 [64], [65]. We quantified TWIST1 expression in a panel of patient derived material, comprising 21 normal prostate samples (adjacent to cancer), 74 primary prostate tumors, of which 9 hormone-refractory samples, and 13 lymph node metastasis. Among the primary tumors are 59 samples of early organ-confined disease, 9 samples of invasive tumors that eventually developed metastasis during follow-up and 6 tumors with lymph node and/or distant metastasis at the time of operation. Quantitative PCR confirmed overexpression of TWIST1 in primary PCa samples and lymph node metastasis (Fig. 5A). In this patient cohort, TWIST1 expression could not predict progression, as it did not differ between non-progressive organ-confined tumors and primary cancers that eventually developed metastasis. Furthermore, TWIST1 expression was not increased in hormone-refractory tumors when compared to hormone-naïve samples, suggesting that TWIST1 overexpression alone may not be enough to confer hormone-refractory growth. However, since TWIST1 is strongly up-regulated in PCa samples it may still be useful as a cancer marker or therapeutical target.

VAV3 is a member of the VAV family of oncoproteins, GTPase guanine nucleotide exchange factors that regulate receptor protein tyrosine kinases. It can be activated upon engagement of growth factor receptors, such as EGFR, PDGFR, INSR or IGF1R, and in turn activate downstream PLC and PI3K signaling pathways [51], [52], [66]. Previous studies have implicated VAV3 in the pathogenesis of the prostate: (i) VAV3 expression has been detected in the prostate, at increased levels in cancer cells [67]; (ii) it has been shown to interact with the AR pathway, stimulating ligand-independent cell growth in LNCAP-hormone-refractory cells [67], [68], and (iii) targeting of constitutively active VAV3 expression to the prostate induced PCa in mice [69]. Surprisingly, in our patient derived samples, VAV3 expression decreased gradually during PCa progression, with the lowest levels in metastatic and hormone-refractory samples (Fig. 5C). Furthermore, Kaplan-Meier analysis showed a direct correlation between VAV3 expression levels and metastasis-free survival (Fig. 5D). These results encourage the use of VAV3 as a potential prognosis marker in PCa, and provide a possible mechanism for the bypass of AR pathway in therapy-refractory tumors. However, the decrease of VAV3 expression in PCa progression was unexpected, considering the function of VAV3 as a potential oncogene. In fact, VAV3 has three transcript variants, the full length VAV3, the VAV3 beta isoform and the truncated VAV3.1 variant, which has no guanine nucleotide exchange activity (GEF) due to lack of N-terminal domains [51], [66], [70]. The truncated transcript is expressed in many tissues and is the major variant in the prostate [51], [70]. Because the effect of VAV3 on cell division and AR activation is dependent on GEF activity, this variant is not oncogenic and has most likely a different function than the full-length protein. It was proposed that this VAV3.1 variant may function as a dominant negative of other VAV family members [51], [70]. In this context, a decrease in the VAV3.1 variant could actually result in increased activity of oncogenic VAV proteins. The TaqMan primers that we used in the quantitative-PCR target the last 2 of the 27 VAV3 exons, being possible that this assay preferentially captures the short C-terminal VAV3.1 transcript. Clearly, it is essential to characterize the different VAV3 variants in the prostate and evaluate how the balance of these is affected during PCa progression, before one can consider its use in the clinic. These results exemplify the limitation of large-scale expression profiling assays that rely on a single probe per gene. Ultimately, to investigate gene expression in the context of human disease, it may not be enough to quantify the major known transcript but one may need to consider the different isoforms and how these variants interact with each other. In the near future we expect to be able to answer how different splice variants from the same gene (including VAV3) can relate to PCa, using exon microarray analysis of the patient tumor material.

DKK3 is part of an evolutionary conserved gene family encoding secreted proteins, which play an important role in vertebrate embryonic development as antagonists of Wnt/beta-catenin signaling. DKKs are further implicated in bone formation and bone disease, Alzheimer's and cancer [71]. DKK3 was proposed to function as a tumor suppressor since it was found to be down-regulated in a number of malignancies including kidney, bladder, lung, pancreas and prostate cancer [72]. Reduced DKK3 expression may, at least in part, be explained by promotor methylation, which has been detected in various cancers, including over 65% of prostate tumors [73]. Additional reports showed consistent reduction of DKK3 expression in prostate adenocarcinomas, particularly those with a high Gleason grade [74], [75]. Moreover, small interfering RNA-mediated down-regulation of DKK3 enhanced cell cycle progression and disrupted three-dimensional acinar morphogenesis in RWPE-1 prostate epithelial cells [74]. Conversely, ectopic expression of DKK3 resulted in decreased proliferation, inhibited colony formation and induced apoptosis of LNCaP, PC3 and DU145 cell lines [73], [74]. In our patient samples, DKK3 expression decreased in prostate cancer and lymph node metastasis, but no difference was observed in hormone-refractory samples (Fig. 5B). As for TWIST1, DKK3 might be useful as a cancer marker, but could not predict tumor progression, nor explain recurrence after hormonal therapy. Interestingly, injection of an adenovirus vector carrying DKK3 showed a dramatic anti-tumor effect in a xenograft human PCa model, inhibiting tumor growth and lymph node metastasis and prolonging mice survival [76]. Such results encourage the development of therapies targeting DKK3 in advanced metastatic disease.

An important limitation of the present study and other transcript profiling studies is that, in most cases, the functional biological entity is the protein, not the measured mRNA. For all of our validated genes of interest (VAV3, TWIST1 and DKK3), it has previously been established that the level of the mRNA expression is representative for its protein level, in pulmonary fibrosis, gastric cancer and various cell lines [68], [77], [78], [79], [80].

In conclusion, the present study shows that AR overexpression (in PC346Flu1) and mutation (in PC346Flu2) may allow for the maintenance of the AR activity under androgen ablation and antiandrogen treatment. In contrast, PCa cells may acquire complete independence from AR signaling by activating alternative survival pathways, as exemplified by PC346DCC. In PC346DCC, activation of VAV3 and TWIST1 oncogenes and down-regulation of DKK3 tumor suppressor constitutes a possible mechanism for bypassing the AR pathway. The fact that TWIST1 and DKK3 expression was deregulated in both hormone-refractory and hormone-naïve patient samples, suggests that these alterations occur earlier in PCa progression and do not act alone in inducing therapy-resistant growth. Indeed, both VAV3 and TWIST1 are known to interact with growth factor signaling, which could be the effector mechanism in stimulating cellular proliferation. Additionally, DKK3 down-regulation may promote survival through inhibition of apoptosis. These results grant further investigations on the use of VAV3, TWIST1 and DKK3 as prostate cancer markers and in the development of targeted therapies for advanced disease.

## Supporting Information

Table S1 Genes differentially expressed in PC346DCC vs. PC346C, expression ratios and SAM q-values.
(0.09 MB XLS)

Table S2 Genes differentially expressed in PC346Flu1 vs. PC346C, expression ratios and SAM q-values.
(0.07 MB XLS)

Table S3 Genes differentially expressed in PC346Flu2 vs. PC346C, expression ratios and SAM q-values.
(0.05 MB XLS)

Table S4 List of androgen-receptor target genes in PC346 cell lines.
(0.04 MB XLS)
